# Systematic Review and Meta-Analysis of L1-VLP-Based Human Papillomavirus Vaccine Efficacy against Anogenital Pre-Cancer in Women with Evidence of Prior HPV Exposure

**DOI:** 10.1371/journal.pone.0090348

**Published:** 2014-03-03

**Authors:** Ada Miltz, Huw Price, Maryam Shahmanesh, Andrew Copas, Richard Gilson

**Affiliations:** Centre for Sexual Health and HIV Research, Research Department of Infection and Population Health, Mortimer Market Centre, University College London, London, United Kingdom; Baylor College of Medicine, United States of America

## Abstract

**Background:**

It is unclear whether L1-VLP-based human papillomavirus (HPV) vaccines are efficacious in reducing the likelihood of anogenital pre-cancer in women with evidence of prior vaccine-type HPV exposure. This study aims to determine whether the combined results of the vaccine trials published to date provide evidence of efficacy compared with control (hepatitis A vaccine/placebo).

**Methods:**

A systematic review and meta-analysis was conducted. Randomized-controlled trials (RCTs) were identified from MEDLINE, Embase, Web of Science, PubMed, Cochrane Central Register of Controlled Trials and references of identified studies. The bivalent vaccine containing HPV-16 and 18 VLPs from GlaxoSmithKline Biologicals (Rixenstart, Belgium), the quadrivalent vaccine containing HPV-6, 11, 16, and 18 VLPs from Merck & Co., Inc., (Whitehouse Station, NJ USA), and the HPV-16 monovalent vaccine from Merck Research Laboratories (West Point, PA USA) were evaluated.

**Findings:**

Three RCT reports and two post-trial cohort studies were eligible, comprising data from 13,482 women who were included in the vaccine studies but had evidence of HPV infection at study entry. Data on efficacy was synthesized using the Mantel-Haenszel weighted fixed-effect approach, or where there was heterogeneity between studies, the DerSimonian and Laird weighted random-effect approach. The mean odds ratio (OR) and 95% confidence interval (CI) for the association between *Cervarix*, *Gardasil* and HPV-16 monovalent vaccine and HPV-associated cervical intraepithelial neoplasia grade 3 or worse was 0·90 (95% CI: 0·56, 1·44). For the association between *Gardasil* and HPV-associated vulval/vaginal intraepithelial neoplasia grades 2–3, the overall OR and 95% CI was 2.25 (95% CI: 0·78, 6.50). Sample size and follow-up were limited.

**Conclusions:**

There was no evidence that HPV vaccines are effective in preventing vaccine-type HPV associated pre-cancer in women with evidence of prior HPV exposure. Small effects of vaccination however cannot be excluded and a longer-term benefit in preventing re-infection remains possible.

## Introduction

Cervical cancer is the second leading cause of cancer-related death in women.[1] Oncogenic human papillomavirus (HPV) plays a critical aetiological role in anogenital cancers. At least 70% of cervical cancers are associated with type 16 or 18.[2] HPV-16 and 18 are also the virus types with which the majority of vulval and vaginal pre-cancer are associated. HPV-16/18 bivalent (*Cervarix*) and HPV-6/11/16/18 quadrivalent (*Gardasil*) vaccines are highly effective in preventing vaccine-type HPV-related genital pre-cancer in women who are HPV-negative at the time of vaccination.[1], [3]

The lifetime risk of a woman acquiring any HPV infection is more than 80%. Half of women acquire cervical infection within 3 years of initiating sexual activity.[4]–[7] About 90% of HPV infections are cleared by the immune system within 6–24 months.[8] The prevalence of HPV infection in sexually active women is 10–20% and even higher in young women.[9], [10] In women who have missed or were not part of adolescent vaccine programmes and who have evidence of HPV exposure (HPV-DNA detected in a cervical sample and/or seropositive for HPV antibody), there is a need to determine the efficacy of prophylactic L1-VLP-based vaccination.

The impact of vaccination on the prevalence of infection in previously exposed women may initially be small, as vaccination may not increase viral clearance.[11]–[13] However, rates of anogenital lesions may decrease over time, if vaccination prevents re-infection.[14]–[18] Evidence for this potential benefit is limited as it is based on small sub-groups of exposed women enrolled in randomized-controlled trials (RCTs) of vaccine efficacy. More precise estimates of vaccine efficacy in women with evidence of prior vaccine-type HPV exposure have not been reported.

There is a need for evidence to inform policy on whether vaccination programmes in older women, who will have a higher proportion with prior exposure, are likely to be of value. The present study aims to determine whether the combined results of the L1-VLP-based HPV vaccine efficacy trials published to date provide evidence that anogenital pre-cancer incidence rates are also reduced in women with evidence of prior vaccine-type HPV exposure. In order to do so, we conducted a systematic review and performed a meta-analysis of published data relating to HPV-exposed women participants. This review was planned to look at vaccine efficacy specifically against HPV-16/18 associated anogenital pre-cancer in women with evidence of prior exposure to types 16 and/or 18. However, it was only possible to analyse vaccine efficacy against vaccine-type HPV-associated anogenital pre-cancer in women with evidence of prior exposure to vaccine and non-vaccine (high-risk) HPV types combined.

## Methods

### Identification of relevant studies

A systematic search was undertaken of MEDLINE, Embase, Web of Science, PubMed, and the Cochrane Central Register of Controlled Trials to identify RCTs of prophylactic HPV vaccination published up to 30 August 2013.

### Inclusion and exclusion criteria; overlap in patient populations across studies

RCT reports and post-RCT follow-on cohort studies published in English that investigated L1-VLP-based vaccine efficacy against vaccine-type HPV-associated cervical intraepithelial neoplasia grade 3 or worse (CIN3+) or vulval/vaginal intraepithelial neoplasia grades 2-3 (VIN2-3/VaIN2-3) were reviewed. Studies providing data on the sub-group of women with evidence of prior vaccine-type HPV exposure, in the total vaccinated cohort (TVC) and the total vaccinated cohort-naïve (TVC-naïve), were eligible for inclusion. The TVC consists of all women who were randomized and received at least one vaccine dose irrespective of baseline HPV-DNA (including those with prevalent anogenital disease), cytological, and serological status. The TVC-naïve consists of all women who received at least one vaccine dose, and were seronegative and HPV-DNA-negative at baseline. Two post-trial cohort studies were included.[15], [19] Three reports were included that presented the results of an analysis of data from multiple trials combined.[1], [14], [20] Combined analyses were included in preference to the component trials [12], [21]–[23] to reduce small-study problems, such as zero cells. Studies published solely in abstract were excluded.

### Outcomes

CIN3 and adenocarcinoma *in situ* (AIS) are lesions with strong malignant potential. About 30% of large CIN3 lesions may progress to cervical cancer within 30 years.[24] The primary outcome was a new diagnosis of CIN3+ (CIN3, AIS, or invasive carcinoma).

VIN3 and VaIN3 are precursors of HPV-related invasive cancers of the vulval and vaginal areas respectively. Due to the reporting in the RCTs of composite endpoints that combined moderate and severe vulval and vaginal lesions, the analysis was planned to use the composite endpoint of incident and persistent prevalence of VIN2-3 or VaIN2-3.

Virological endpoints are prone to misclassification bias in women with evidence of prior HPV exposure. It is difficult to distinguish a re-infection in DNA-negative women, regardless of serostatus, from a ‘reactivation’ of previously undetectable, low-level viral persistence in basal cells. In addition, if seroconversion is detected in vaccinated women, who were DNA-positive but seronegative at baseline, the presence of antibodies could be due to a re-infection, delayed seroconversion to the original infection or response to vaccine. The analysis plan therefore excluded virological endpoints.

### Data extraction

Two independent reviewers (AM and HP) extracted data on trial design, inclusion criteria, participant characteristics, vaccines administered, endpoints, efficacy populations, and methodological quality.

### Statistical analysis

The HPV vaccine trials were not designed to determine efficacy in women with evidence of prior HPV exposure, those that were DNA and/or seropositive at baseline. However, such women were still included as it was not practical to test all potential participants at screening and prior to randomization. One published analysis that combined the results of RCTs that evaluated vaccine efficacy in a sub-group of exposed women (seropositive but HPV-DNA-negative) was eligible for inclusion.[14] The remaining published analyses combining the results of RCTs and post-RCT follow-on cohort studies included in this review did not present data specifically on the outcome in a previously exposed population.[1], [15], [19], [20] However, it was possible to derive this from the reported data, by subtracting the results for TVC-naïve from the TVC. [1], [15], [19], [20]

One post-RCT follow-on cohort study analysed a population of women defined as naïve to the relevant HPV type (NRT).[15] Like the TVC-naïve, this population consisted of subjects who received at least one dose of vaccine or placebo and returned for follow-up. However, instead of excluding women with evidence of exposure to all high-risk or vaccine HPV types, the NRT population excluded only women who were DNA and/or seropositive at enrolment for the HPV type of interest. Among women evaluated for vaccine efficacy against HPV-16/18 associated anogenital pre-cancer, it was possible to derive a sub-group of women with evidence of prior exposure (according to our criteria) to high-risk HPV types 16 and/or 18. It was not possible to do so for the remaining studies. The number of evaluable women in the TVC [1], [19], [20] and the sub-group [14] who were exposed to types 16 and/or 18 at baseline and their corresponding outcome data were not reported. Therefore, vaccine efficacy against vaccine-type HPV-associated anogenital pre-cancer in women with evidence of prior exposure to a range of low- to high-risk HPV types was analysed.

Women from three trials, who were seropositive for one or more of HPV-6/11/16/18 but DNA-negative at baseline, contributed data twice to the CIN3+ and the VIN2-3/VaIN2-3 analysis. [12], [21], [23] In both analyses, the combined analysis that included women who were DNA-positive at baseline reported no information on the number of evaluable women in the TVC who were seropositive and DNA-negative at baseline and their corresponding outcome data.[1], [20]. It was, therefore, not possible to remove these ‘double counted’ participants. In the quadrivalent HPV vaccine clinical program, 73% of women were vaccine- type HPV-naïve at baseline.[12], [21], [23] Of the remaining women, 15% were seropositive and DNA-negative.[14] We estimated that 109 women and 96 women were analysed in two combined analyses for CIN3+ [1], [14] and VIN2-3/VaIN2-3 [14], [20], respectively. The effect of ‘double counting’ patients was likely to be minimal and all three combined analyses were included in this review.

Effect sizes were summarized as odds ratios (ORs) with associated 95% confidence intervals (CIs). The event rates in the vaccine and control arms are displayed as a L'Abbé plot. *X^2^* homogeneity p-values and *I^2^* statistics were used to formally assess heterogeneity across ORs using a Mantel-Haenszel weighted fixed-effect model.[25]–[27] The overall effect was not calculated as potentially different effects were expected with respect to the two outcomes and since many patients contributed data for both outcomes. A p-value of less than 0.1 (rather than 0.05) was considered to indicate heterogeneity due to the small number of studies under investigation. The percentage of between-study heterogeneity was considered low if 25-50%, moderate if 50-75%, and high if 75% and greater.[25] With so few publications under review it was considered unlikely that a formal investigation of heterogeneity would produce useful explanations for variable vaccine effects. The possibility of publication and related bias was formally assessed using a modified Galbraith plot for Harbord's modified test.[28], [29]

Resulting heterogeneity between studies that presented data on CIN3+ was allowed for by incorporating a DerSimonian and Laird weighted random-effects model, since the true vaccine effects were likely to be different but qualitatively similar across studies. This systematic review was performed according to the methods recommended by the Cochrane Collaboration.[30] All analyses were performed using STATA statistical software [31] and reported according to the PRISMA guidelines (Checklist S1).[32]

## Results

### I. Identification and selection of RCT reports and post-RCT follow-on cohort studies

**Figure 1:**
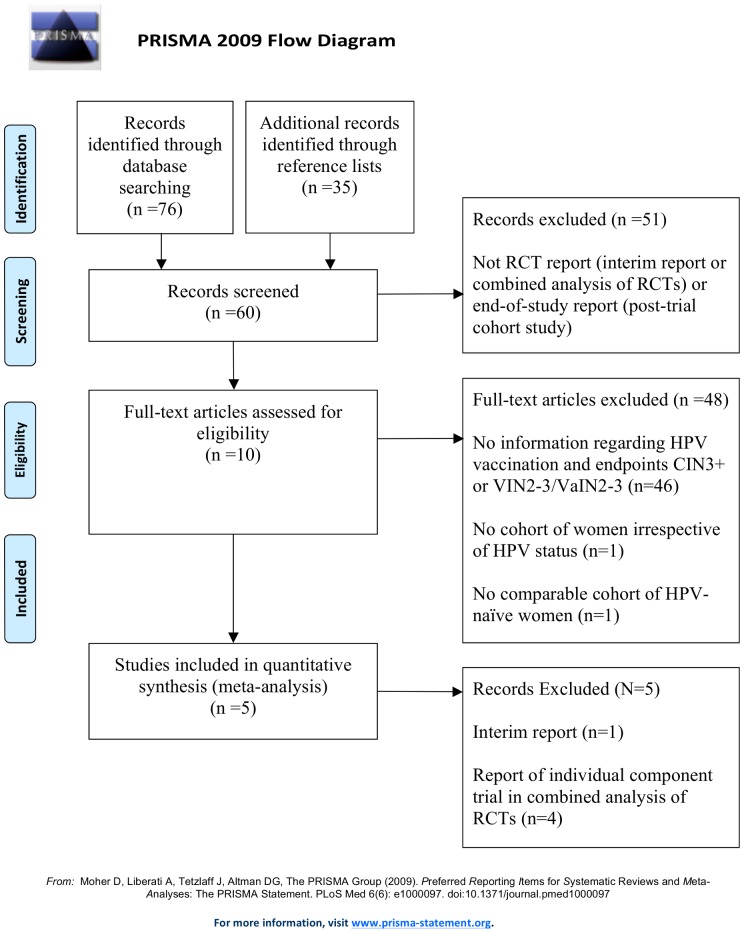
Inclusion and exclusion of publications in selection of RCT reports and post-RCT follow-on cohort studies.

The study search strategy is detailed in Table 1. Of 111 publications identified through a search of databases and reference lists, 106 were excluded for reasons summarised in Figure 1.

**Table 1:** Search strategy and identification of publications.

Database	MeSH terms	Search strategy	Limits	Number of publications identified for systematic review	Number of RCT reports/ post-trial cohort studies
MEDLINE	“HPV” map-term and “papillomavirus infections” or “human papillomavirus* or HPV” non map-term, “Cervarix” map-term and “papillomavirus vaccines” or “bivalent L1 virus-like particle vaccine* or Cervarix” non map-term, “Gardasil” map-term and “papillomavirus vaccines” or “quadrivalent L1 virus-like particle vaccine* or Gardasil” non map-term and “immune response” map-term and “antibody formation” or “immunogenic* or immune response” non map-term.	HPV, immune response & *Cervarix* terms or HPV, immune response & *Gardasil* terms	English language, humans, females, Ovid full text*, and RCTs	22	9
Embase	“HPV” map-term or “human papillomavirus* or HPV” non map-term, “Cervarix” map-term or “bivalent L1 virus-like particle vaccine* or Cervarix” non map-term, “Gardasil” map-term or “quadrivalent L1 virus-like particle vaccine* or Gardasil” non map-term and “immune response” map-term or “immunogenic* or immune response” non map-term	HPV, immune response & *Cervarix* terms or HPV, immune response & *Gardasil* terms	English language, humans, females, Ovid full text*, and RCTs	6	3
Web of Science	“human papillomavirus* or HPV”, “immunogenic* or immune response”, “quadrivalent L1 virus-like particle vaccine* Gardasil” and “bivalent L1 virus-like particle vaccine* Cervarix” “mice* or mouse” and “male* or men”	HPV, immune response & *Cervarix* terms or HPV, immune response & *Gardasil* terms	English language, humans, females, and RCTs	26	8
PubMed	“HPV Cervarix immune response” and “HPV Gardasil immune response”	Two separate searches	Humans, females, English language, links to free full text*, and RCTs	12 (6 *Cervarix* and 6 *Gardasil*)	8 (3 *Cervarix* and 5 *Gardasil*)
Cochrane Central Register of Controlled Trials	“human papillomavirus”, “immune response” and “Gardasil” or “human papillomavirus”, “immune response” and “Quadrivalent L1 virus-like particle vaccine” “mice” and “male”	Combination of vaccine names in four separate searches	Humans and females	10	10 (2 *Cervarix* and 8 *Gardasil*/quadrivalent vaccine)
Reference lists	Additional relevant publications were identified through hand search of bibliographies of publications obtained through the MeSH term searches	/	/	35	22

*When full text was not imposed as a limitation, no additional RCT reports or post-RCT follow-on cohort studies were eligible for the meta-analysis, identified via abstracts.

### II. Characteristics of included RCT reports and post-RCT follow-on cohort studies, and trial participants (Table 2)

**Figure 2:**
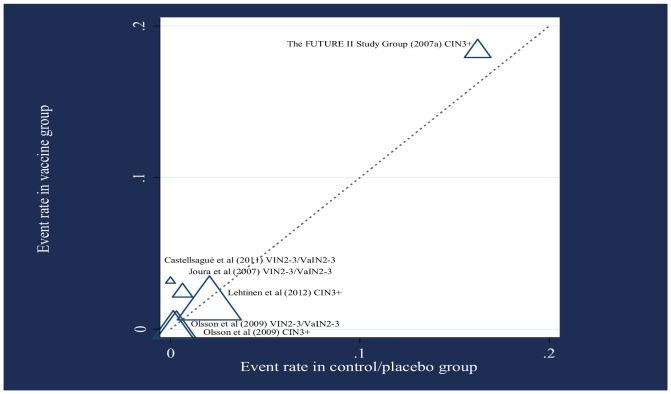
L'Abbé plot displaying the rate of cervical or vulval/vaginal lesions at end-of-study follow-up. Symbol size represents sample size. Results are displayed in terms of the line of equality where event rate in vaccine group  =  event rate in control/placebo group

The methodological quality was high for the studies determined as eligible. Each demonstrated adequate reporting of allocation concealment, blinding and dropouts, and loss-to-follow-up. The longest follow-up period was 4 years. The event rates in the vaccine and control/placebo arms are displayed in Figure 2.

**Table 2:** Summary of selected characteristics of three RCT reports and two post-RCT follow-on cohort studies contributing to a meta-analysis.

Author	Lehtinen et al 2012[19]	The FUTURE II Study Group 2007a[1]	Castellsagué et al 2011[15]	Joura et al 2007[20]	Olsson et al 2009[14]
Study design	Post-RCT follow-on cohort study	Combined analysis of four RCTs [12], [21]–[23]	Post-RCT follow-on cohort study	Combined analysis of three RCTs [12], [21], [23]	Combined analysis of three RCTs [12], [21], [23]
**Mean (SD) age of participants in vaccine arm**	20(3.1)	20(2)	34.3(6.3)	20(2)	20.7(1.8)
**Mean (SD) age of participants in control/placebo arm**	20(3.1)	20(2)	34.3(6.3)	20(2)	20.6(1.9)
**Inclusion criteria for number of lifetime sexual partners** *	6 or fewer	4–5 or fewer	N/A****	4 or fewer	4 or fewer
**Median lifetime number of sexual partners in vaccine arm**	No data**	2	2	3	3
**Median lifetime number of sexual partners in control/placebo arm**	No data**	2	2	3	3
**Years of follow-up**	4	3	3.8	3	3
**HPV vaccine: comparator**	*Cervarix*: hepatitis A vaccination	*Gardasil*/HPV-16 monovalent vaccine: placebo***	*Gardasil*: placebo	*Gardasil*: placebo	*Gardasil*: placebo
**Women were evaluated for vaccine efficacy against endpoints associated with HPV type(s):**	16/18	16/18	16/18	16/18	6/11/16/18
**Histological endpoint**	CIN3+	CIN3+	VIN2 or 3 or VaIN2 or 3	VIN2 or 3 or VaIN2 or 3	CIN3+ and VIN2 or 3 or VaIN2 or 3

*In accordance with local regulatory and ethical requirements, an exclusion criterion for number of lifetime sexual partners was not applied in Finland. As a result, a proportion of 16–17 year old Finnish girls had more than 4,[14], [20] 5,[1] or 6 [19] lifetime partners.

**The post-PATRICIA trial cohort study did not publish the median lifetime number of sexual partners of study participants.[19] Data regarding the number of sexual partners in the past 12 months (at baseline) was available; the median number of sexual partners in the past year was 1 in both the vaccine and control group arms of the study.

***Women from the HPV-16 monovalent vaccine trial did not contribute to analysis of HPV-18 related endpoints.[22].

****Lifetime number of sexual partners was not an inclusion or exclusion criterion.[15].

### III. Infection and disease characteristics of women with evidence of prior exposure (Table 3)

In this review the women evaluated for vaccine efficacy were a sub-group of the women enrolled in the TVC.

### IV. Heterogeneity

There was significant heterogeneity (p = 0·08; *I^2^* = 60%) between studies presenting data on CIN3+, and on inspection of the effect measures we interpret this to indicate that the effect of vaccination probably differed between the combined analyses and post-RCT follow-on cohort study. Exposure and disease status differed across the sub-groups of exposed women in these studies. In the FUTURE trials women with HPV-associated disease at baseline were included.[12], [23] By not using a pre-planned cohort of exposed women it was possible that heterogeneity was present across the pooled combined analyses and post-RCT follow-on cohort study due to lower efficacy found in the combined analysis that incorporated women with prevalent cervical disease at baseline (Table 3).[1] However, we did not formally investigate the extent to which statistical heterogeneity between results of the studies was related to the inclusion of women with HPV-associated disease.

**Table 3:** Women with evidence of prior exposure evaluated for vaccine efficacy.

	Lehtinen et al (2012)[19]	The FUTURE II Study Group (2007a)[1]	Castellsagué et al 2011[15]	Joura et al (2007)[20]	Olsson et al (2009)[14]
**TVC** * **included:**		Combination of women enrolled in the TVC** of four individual trials:[12], [21]–[23]		Combination of women enrolled in the TVC** of three individual trials:[12], [21], [23]	Sub-population of 2617 women from three individual trials who were seropositive and DNA-negative to one or more vaccine HPV type(s) at day 1.[12], [21], [23]
HPV-naïve	¶	¶	¶	¶	
Evidence of exposure to any high-risk or low-risk HPV type	¶	¶	¶	¶	
Abnormal cervical cytology	¶	¶		¶	
Prevalent cervical disease		¶			
Prevalent anogenital disease				¶	
**TVC-naïve included:**		Combination of women enrolled in the TVC-naïve of four individual trials:[12], [21]–[23]		Combination of women enrolled in the TVC-naïve of three individual trials:[12], [21], [23]	
HPV-naïve	¶	¶		¶	
Evidence of current exposure to any low-risk HPV type	¶				
Evidence of past exposure to any non-vaccine HPV type	¶				
Evidence of exposure to any non-vaccine HPV type		¶		¶	
Abnormal cervical cytology		¶		¶	
**NRT for HPV-16/18 related outcomes included:**					
HPV-naïve			¶		
Evidence of exposure to vaccine types 6/11 and any non-vaccine HPV type			¶		
**Derived sub-group of exposed women included:**					
HPV-16/18/31/33/35/39/45/51/52/56/58/59/66/68 DNA+ (and potentially sero+) or HPV-16/18 sero+	¶				
HPV-6/11/16/18 DNA+ and/or sero+		¶		¶	
HPV-16/18 DNA+ and/or sero+			¶		
Abnormal cervical cytology	¶				
Prevalent cervical disease		¶			
Prevalent anogenital disease				¶	
**Number of women in derived sub-group:**	6484	1117	143	643	

*Women with a history of genital warts or warts at baseline were not included in the TVC.

**A TVC was not investigated in one individual RCT.[21] Outcomes for a TVC in this report were derived from the summary of participants excluded from analysis, extracting data specifically on women excluded from the TVC-naïve.

### V. Small-study effects (Figures 3 & 4)

The modified Galbraith plot and Harbord's test (CIN3+, p = 0·37; VIN2-3/VaIN2-3, p = 0.76) indicated no significant publication and related bias.

**Figure 3:**
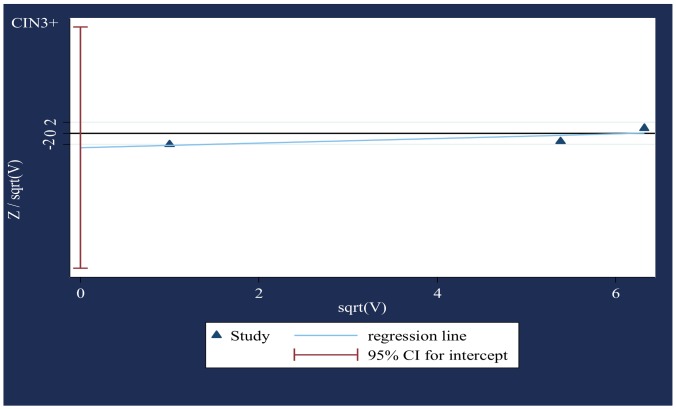
Modified Galbraith plot for studies that presented data on CIN3+ (based on two-sided p-values). Standard normal deviate of OR against its precision.

**Figure 4:**
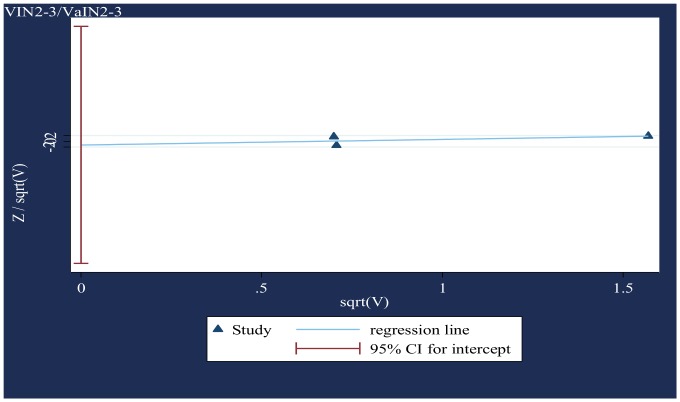
Modified Galbraith plot for studies that presented data on VIN2-3/VaIN2-3 (based on two-sided p-values). Standard normal deviate of OR against its precision.

### VI. Outcomes (Table S1)

**Figure 5:**
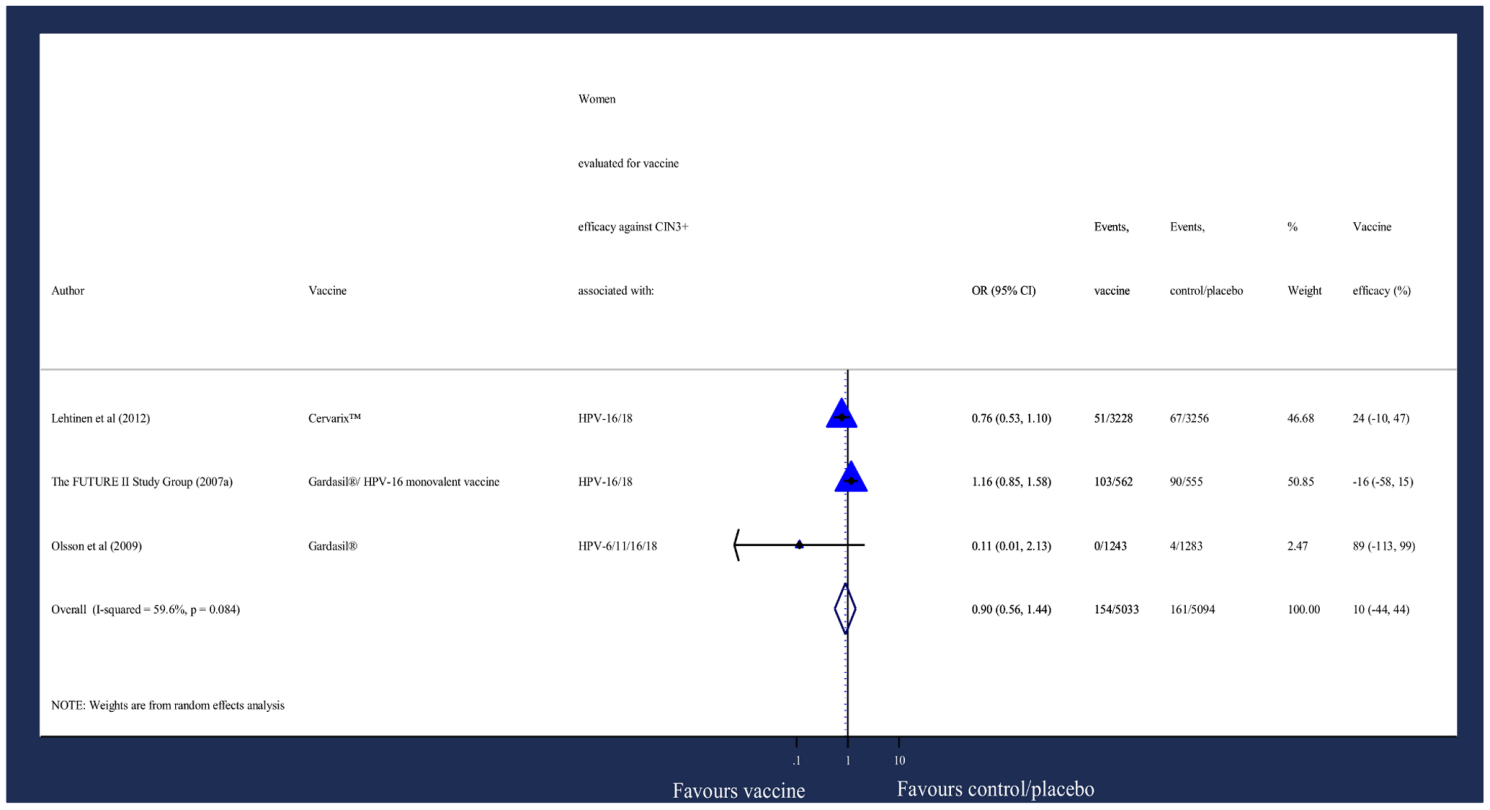
Odds of new diagnosis of CIN3+.

**Figure 6:**
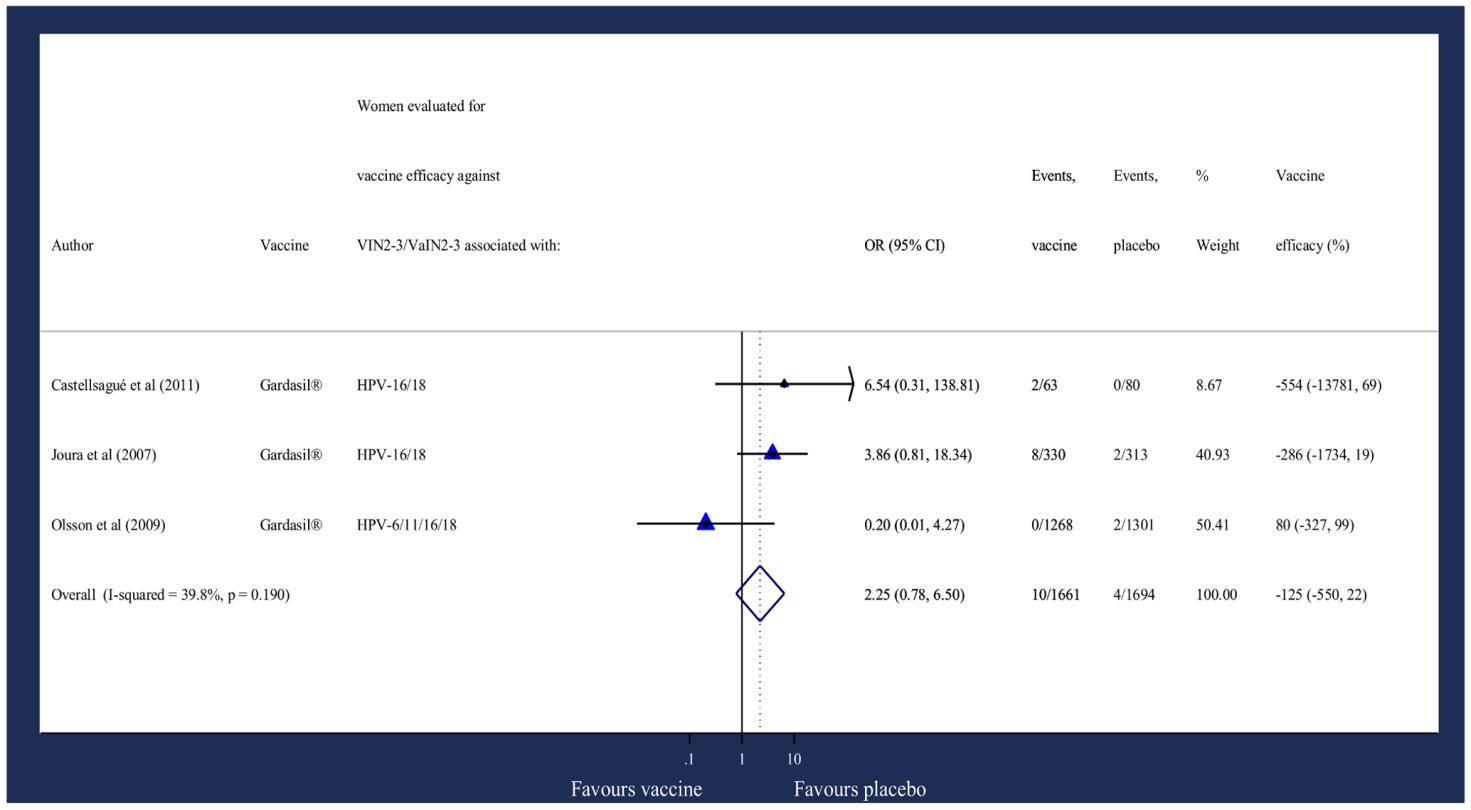
Odds of incident/persistent prevalence of composite vulval/vaginal lesions.

1. CIN3+ (Figure 5)
2.
  - The DerSimonian and Laird weighted mean OR for 10,127 women with evidence of prior HPV exposure was 0·90 (95% CI: 0·56, 1·44).[1], [14], [19] The corresponding pooled efficacy was 10% (95% CI: -44, 44).
3. VIN2-3/VaIN2-3 (Figure 6)
4.
  - The Mantel-Haenszel weighted overall OR for 3355 women with evidence of prior vaccine-type HPV exposure was 2.25 (95% CI: 0·78, 6.50).[14], [20] The corresponding pooled efficacy was -125% (95% CI: -550, 22).

There was no statistically significant reduction in CIN3+ or VIN2-3/VaIN2-3 incidence for vaccine recipients compared with control/placebo recipients.

## Discussion

There was no evidence from this analysis that HPV vaccines given to women with evidence of prior HPV infection can prevent vaccine-type HPV-associated CIN3+ and VIN2-3 or VaIN2-3. However, there are several limitations to this review. The trials included were not designed or powered to evaluate the effect of vaccination in women with evidence of prior vaccine-type HPV exposure. There have been no RCTs to date that have been designed to investigate the effect of vaccination in previously exposed women. For example, the PATRICIA trial was designed to assess vaccine efficacy in women who were HPV-DNA-negative and seronegative at baseline.[33] However, previously exposed women (DNA and/or seropositive) were not excluded at enrolment, and an analysis of this group has suggested that there is protection against re-infection. Castellsagué et al. reported a 66.9% (95% CI: 4.3, 90.6) quadrivalent vaccine efficacy against vaccine-type persistent infection (there were no cases of CIN or external genital lesions) in women seropositive and DNA-negative for HPV-6/11/16/18.[15] Among women who were DNA-negative and seropositive for HPV-16/18, bivalent vaccine efficacy against vaccine-type infection that was persistent for at least 6-months, was 72.3% (95% CI: 53.0, 84.5).[16] However, the statistical power of these sub-group analyses is low. In the PATRICIA trial, less than 20% of women in the TVC were eligible for inclusion in this analysis.

The endpoints investigated in this review were rare. The annual event rate is 0·19% for HPV-16 related and 0·038% for HPV-18 related cervical pre-cancer. VIN2-3/VaIN2-3 lesions were even less frequent.[34]–[37] Even combining sub-groups of women (with all sub-categories of exposure i.e. DNA and/or seropositive) in this meta-analysis did not provide sufficient events to be sure of identifying a modest difference between the vaccine and control/placebo group arms over 3–4 years.

This meta-analysis was not able to look at the efficacy of vaccination on virological endpoints in previously exposed women. The efficacy of vaccine to prevent re-infection in seropositive and DNA-negative women, or following transient infection in DNA-positive women over 3–4 years is an important question. However, the absence of a reliable method to distinguish vaccine from natural antibody precluded us from investigating it in this meta-analysis.

**Figure 7:**
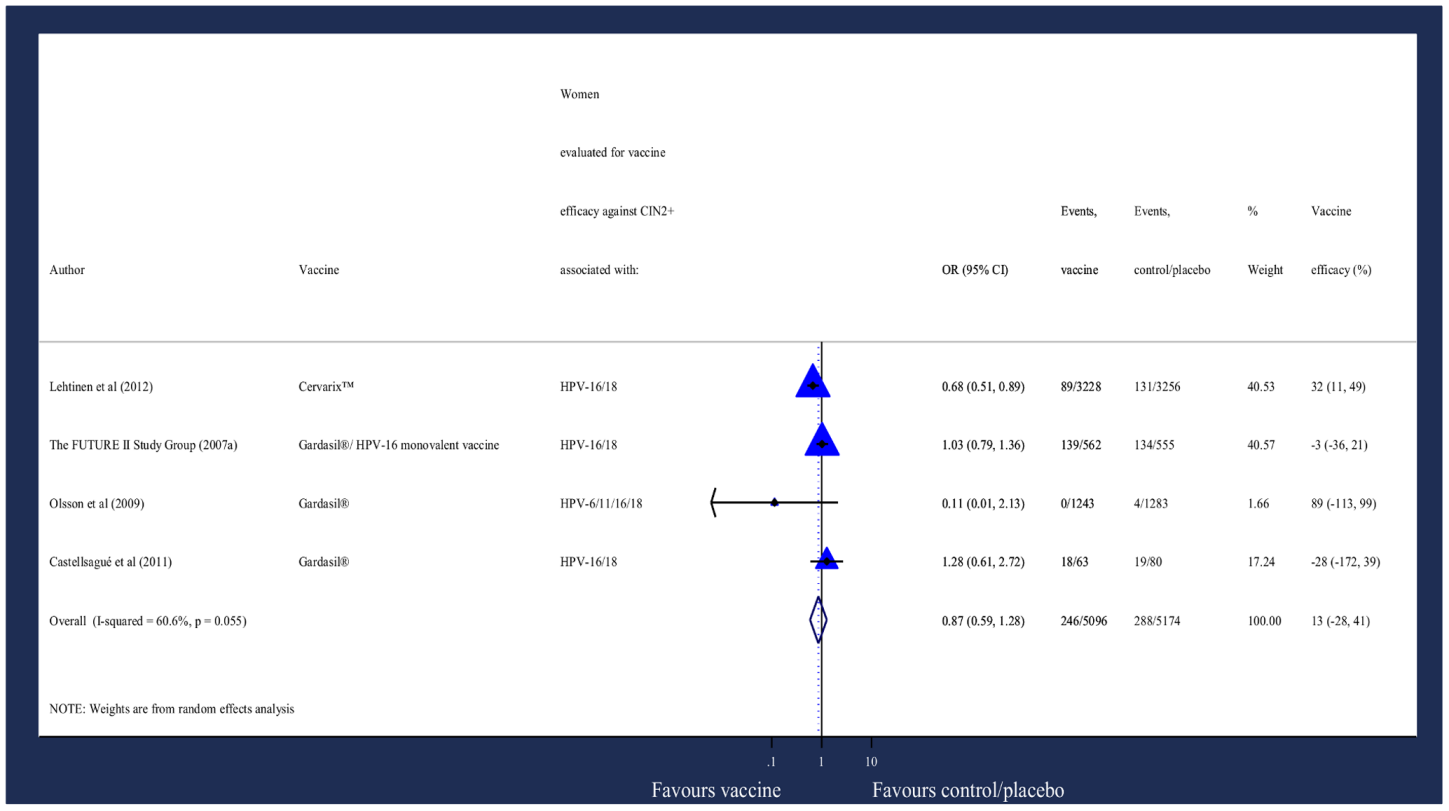
Odds of new diagnosis of CIN2+.

Due to the design of the studies included, it was also possible to analyse CIN grade 2 or worse (CIN2+) as the endpoint, which would increase the number of events over 3–4 years (Table S2). However CIN2+ is not as strongly associated with high-risk HPV types.[1], [19] The analysis showed no statistically significant reduction in CIN2+ incidence in vaccine recipients compared with control/placebo recipients (Figure 7).

An analysis of women with HPV-associated anogenital disease in the TVC has suggested that vaccine provides protection against recurrent disease. Among all women who underwent definitive cervical therapy in the FUTURE I and II studies, quadrivalent vaccination was associated with a 64.9% (95% CI: 20.1%, 86.3%) reduction in the rate of any subsequent CIN2+.[18] A recent study investigated whether quadrivalent vaccination after treatment with loop electrosurgical excision for CIN2-3 is effective in preventing recurrent disease. Among patients who had CIN2-3 associated with vaccine HPV types, the control group had a significantly higher recurrence rate than the vaccination group (8.5% and 2.5% respectively); p<0.05).[17]. Although HPV vaccination trials did not include HPV testing (DNA or serology) or clinical examination before randomization, women with a prior history of HPV-related disease were excluded from enrolment. Therefore, trials conducted to date cannot measure the efficacy of the vaccine in women who have undergone treatment before vaccination to investigate a potential reduction in the risk of any subsequent high-grade disease. There is a need to design trials to answer this question.

Furthermore, pre-cancer typically takes 5–10 years to develop from incident infection,[35]–[37] whereas the follow-up available in the combined analyses and post-RCT follow-on cohort studies was 3-4 years. If a second incident infection with the same HPV type as the women had been exposed to at baseline did occur it could still take many years before women experienced precancerous lesions. This would suggest that the duration of follow-up available in the combined analyses and post-RCT follow-on cohort studies in this meta-analysis is too short to detect an effect of HPV vaccination on disease from re-infection in HPV-exposed women. Whether vaccination will provide long-term benefit in this sub-group by protecting against re-infections and subsequent disease will require further investigations with longer follow-up.

Finally, not all women in the VIN2-3/VaIN2-3 analysis were evaluated for vaccine efficacy against VIN2-3/VaIN2-3 associated with one or more of the types to which they were exposed (HPV-6/11/16/18). Women from one combined analysis who had evidence of HPV-6/11 exposure prior to vaccination would not have been at risk for HPV-16/18 associated VIN2-3/VaIN2-3.[20] In the CIN3+ analysis women in two combined analyses who had evidence of HPV-6/11 exposure prior to vaccination were included.[1], [14] Women from one of the combined analyses were evaluated for vaccine efficacy against CIN3+ associated with HPV-6/11/16/18. However types 6 and 11 do not cause CIN3+. In addition, women from the post-RCT follow-on cohort study who had evidence of current infection with high-risk HPV types other than HPV-16/18 (HPV-31/33/35/39/45/51/52/56/58/59/66/68) prior to vaccination were included.[19] These women would not have been at risk for HPV-16/18 associated CIN3+.[1], [14], [19]

Therefore, this review was a refinement of the TVC in terms of exposed women, but it did not conclusively assess the effect of L1-VLP-based vaccination against vaccine-type HPV-associated anogenital pre-cancer in women who had been at risk for the endpoint prior to vaccination. Moreover, some patients (though proportionately not many) were double counted in the meta-analysis. Finally, the estimation of variance can be imprecise in a DerSimonian and Laird model with very small numbers of included publications. Findings should be interpreted with caution.

## Conclusion

There was no evidence from this analysis that HPV vaccines given to women with evidence of prior vaccine-type HPV exposure can prevent premalignant lesions related to these HPV types over a 3–4 year time frame. Given the number of events, a small effect of vaccination however cannot be excluded and this review could not address the issue of efficacy against recurrent infection. Further studies are warranted to investigate these issues.

## Supporting Information

Table S1 **Distribution of odds in three eligible RCT reports and two post-RCT follow-on cohort studies.** * Data for CIN3 and AIS was reported separately. The number of evaluable women and cases where combined to calculate ORs for CIN3+. **Number of evaluable women with evidence of prior exposure reporting at least one event. ‡Number of evaluable women with evidence of prior exposure not reporting an event.‡‡An OR less than 1 suggested vaccine protection. ¥Vaccine efficacy was estimated as 100x (1-OR) and expressed as the percentage reduction in odds of CIN3+ or VIN2-3/VaIN2-3 compared to the control/placebo.
(DOC)

Table S2 **Distribution of odds in four studies. Results displayed in terms of vaccine efficacy against CIN2+.** *Number of evaluable women with evidence of prior exposure reporting at least one event. ‡Number of evaluable women with evidence of prior exposure not reporting an event. ‡‡An OR less than 1 suggested vaccine protection. ¥Vaccine efficacy was estimated as 100x (1-OR) and expressed as the percentage reduction in odds of CIN2+ compared to the control/placebo.
(DOC)

Checklist S1 **PRISMA 2009 checklist.**
(DOC)
