# Supplementary material for: Systematic Review and Meta-Analysis of L1-VLP-Based Human Papillomavirus Vaccine Efficacy against Anogenital Pre-Cancer in Women with Evidence of Prior HPV Exposure
Source: PLoS One. 2014 Mar 3;9(3):e90348. doi: 10.1371/journal.pone.0090348 (PMC3940851; doi:10.1371/journal.pone.0090348)
Supplement: Table S1 — Distribution of odds in three eligible RCT reports and two post-RCT follow-on cohort studies. * Data for CIN3 and AIS was reported separately. The number of evaluable women and cases where combined to calculate ORs for CIN3+. **Number of evaluable women with evidence of prior exposure reporting at least one event. ‡Number of evaluable women with evidence of prior exposure not reporting an event.‡‡An OR less than 1 suggested vaccine protection. ¥Vaccine efficacy was estimated as 100x (1-OR) and expressed as the percentage reduction in odds of CIN3+ or VIN2-3/VaIN2-3 compared to the control/placebo. (DOC) [file pone.0090348.s001.doc]

| **Author** | **HPV vaccine** | **Histological endpoint** | **Women were evaluated for vaccine efficacy against endpoints associated with HPV type(s):** | **No. of evaluable women with evidence of prior exposure in vaccine arm** | **No. of evaluable women with evidence of prior exposure in control/placebo arm** | **Cases in vaccine arm**** | **Cases in control/placebo arm**** | **Non-cases in vaccine arm‡** | **Non-cases in control/placebo arm‡** | **Odds ratio (95% CI)‡‡** | **Vaccine efficacy (95%CI)**¥ |
| --- | --- | --- | --- | --- | --- | --- | --- | --- | --- | --- | --- |
| Lehtinen et al (2012) | *Cervarix* | CIN3+ | 16/18 | 3228 | 3256 | 51 | 67 | 3177 | 3189 | 0·76 (0·53, 1·10) | 24 (-10, 47) |
| The FUTURE II Study Group (2007a) | *Gardasil*/HPV-16 monovalent vaccine | CIN3+* | 16/18 | 562 | 555 | 103 | 90 | 459 | 465 | 1·16 (0·85, 1·58) | -16 (-58, 15) |
| Olsson et al (2009) | *Gardasil* | CIN3+ | 6/11/16/18 | 1243 | 1283 | 0 | 4 | 1243 | 1279 | 0·11 (0·01, 2·13) | 89 (-113, 99) |
| DerSimonian and Laird weighted mean effect | *Cervarix, Gardasil* & HPV-16 monovalent vaccine | CIN3+ | 6/11/16/18 | 5033 | 5094 | 154 | 161 | 4879 | 4933 | 0·90 (0·56, 1·44) | 10 (-44, 44) |
| Castellsagué et al (2011) | *Gardasil* | VIN2-3 or  VaIN2-3 | 16/18 | 63 | 80 | 2 | 0 | 61 | 80 | 6.54 (0.31, 138.81) | -554 (-13781, 69) |
| Joura et al  (2007) | *Gardasil* | VIN2-3 or  VaIN2-3 | 16/18 | 330 | 313 | 8 | 2 | 322 | 311 | 3·86 (0·81, 18·34) | -286 (-1734, 19) |
| Olsson et al (2009) | *Gardasil* | VIN2-3 or  VaIN2-3 | 6/11/16/18 | 1268 | 1301 | 0 | 2 | 1268 | 1299 | 0·20 (0·01, 4·27) | 80 (-327, 99) |
| Mantel-Haenszel weighted overall effect | *Gardasil* | VIN2-3 or  VaIN2-3 | 6/11/16/18 | 1661 | 1694 | 10 | 4 | 1651 | 1690 | 2.25 (0.78, 6.50) | -125 (-550, 22) |
