# Supplementary material for: Systematic Review and Meta-Analysis of L1-VLP-Based Human Papillomavirus Vaccine Efficacy against Anogenital Pre-Cancer in Women with Evidence of Prior HPV Exposure
Source: PLoS One. 2014 Mar 3;9(3):e90348. doi: 10.1371/journal.pone.0090348 (PMC3940851; doi:10.1371/journal.pone.0090348)
Supplement: Table S2 — Distribution of odds in four studies. Results displayed in terms of vaccine efficacy against CIN2+. *Number of evaluable women with evidence of prior exposure reporting at least one event. ‡Number of evaluable women with evidence of prior exposure not reporting an event. ‡‡An OR less than 1 suggested vaccine protection. ¥Vaccine efficacy was estimated as 100x (1-OR) and expressed as the percentage reduction in odds of CIN2+ compared to the control/placebo. (DOC) [file pone.0090348.s002.doc]

| **Author** | **HPV vaccine** | **Histological endpoint** | **Women were evaluated for vaccine efficacy against endpoints associated with HPV type(s):** | **No. of evaluable women with evidence of prior exposure in vaccine arm** | **No. of evaluable women with evidence of prior exposure in control/placebo arm** | **Cases in vaccine arm*** | **Cases in control/placebo arm*** | **Non-cases in vaccine arm‡** | **Non-cases in control/placebo arm‡** | **Odds ratio (95%CI)‡‡** | **Vaccine efficacy (95%CI)**¥ |
| --- | --- | --- | --- | --- | --- | --- | --- | --- | --- | --- | --- |
| Lehtinen et al (2012) | *Cervarix* | CIN2+ | 16/18 | 3228 | 3256 | 89 | 131 | 3139 | 3125 | 0·68 (0·51, 0·89) | 32 (11, 49) |
| The FUTURE II Study Group (2007a) | *Gardasil*/HPV-16 monovalent vaccine | CIN2+ | 16/18 | 562 | 555 | 139 | 134 | 423 | 421 | 1·03 (0·79, 1·36) | -3 (-36, 21) |
| Olsson et al (2009) | *Gardasil* | CIN2+ | 6/11/16/18 | 1243 | 1283 | 0 | 4 | 1243 | 1279 | 0·11 (0·01, 2·13) | 89 (-113, 99) |
| Castellsagué et al (2011) | *Gardasil* | CIN2+ | 16/18 | 63 | 80 | 18 | 19 | 45 | 61 | 1.28 (0.61, 2.72) | -28 (-172, 39) |
| DerSimonian and Laird weighted mean effect | *Cervarix, Gardasil* & HPV-16 monovalent vaccine | CIN2+ | 6/11/16/18 | 5096 | 5174 | 246 | 288 | 4850 | 4886 | 0.87 (0.59, 1.28) | 13 (-28, 41) |
